# Effects of Peanuts and Pistachios on Gut Microbiota and Metabolic Syndrome: A Review

**DOI:** 10.3390/foods12244440

**Published:** 2023-12-11

**Authors:** Stéphani Borges Campos, Josemar Gonçalves de Oliveira Filho, Mateus Kawata Salgaço, Marisa Helena De Jesus, Mariana Buranelo Egea

**Affiliations:** 1 Goiano Federal Institute, Campus Rio Verde, Rio Verde 75901-970, Brazil; stephani_bc@yahoo.com.br (S.B.C.); marisahj.01@hotmail.com (M.H.D.J.); 2 School of Pharmaceutical Sciences, São Paulo State University (UNESP), Araraquara 14800-903, Brazil; josemar.gooliver@gmail.com (J.G.d.O.F.); mateus.salgaco@unesp.br (M.K.S.)

**Keywords:** dietary impact, functional food, prebiotics, gut health

## Abstract

There is growing evidence that the gut microbiota is associated with various aspects of human health, including immune system regulation, vitamin synthesis, short-chain fatty acid production, etc. Peanuts and pistachios are foods rich in protein, unsaturated fatty acids, vitamins, polyphenols, and other dietary components that have been shown to benefit the gut microbiota. Therefore, this review aims to describe the effects of consuming peanuts and pistachios on the gut microbiota and the potential role of these microbiota in human health. This review suggests that the consumption of peanuts or pistachios can demonstrate the potential to exert a beneficial effect on the gut microbiota by promoting the growth of beneficial gut bacteria that produce, for example, short-chain fatty acids that are beneficial for human health. In the case of peanuts, in particular, the possible modulation of the microbiota is associated with an improvement in the risk factors of metabolic syndrome and the inflammatory process triggered by a high-fat diet.

## 1. Introduction

The human body harbors a large number of microorganisms that constitute its particular environment, and the role of these microorganisms in human health has been much discussed in the literature. Therefore, it is essential to understand two specific terms: (i) the microbiome, which refers to the collection of genomes of all microorganisms in the environment; this environment can be the human body itself or specific parts of the human body, such as the intestine, skin, etc., and (ii) the microbiota, which comprises the organisms found in a specific environment, such as microorganisms living on the skin, along the gastrointestinal tract (GIT), and in the intestine (known as the gut microbiota) [1]. In simpler terms, the microbiome is like a library containing the genetic blueprints of all the microorganisms in a place (like our body). At the same time, the microbiota is the natural community of living microorganisms in that place. The microbiota composition depends on the quality of life, physical activity, diet, and other environmental factors, so each individual’s qualitative and quantitative microorganisms are different [2].

The human intestine harbors a variety of microorganisms that play a fundamental role in host development and physiology. Likewise, the composition of the gut microbiota is characterized by interindividual variability, which may be shaped by various factors, such as age, genetics, the mode of birth, the type of infant feeding, medications, geographic region, and diet [2,3]. The gut microbiota is generally varied, mainly composed of the phylum Bacillota (former Firmicutes) (Σ64%), including the genera *Lactobacillus*, *Bacillus*, *Clostridium*, *Enterococcus*, *Ruminococcus*, *Eubacterium*, *Faecalibacterium*, and *Roseburiam*. The second most prevalent phylum is the Bacteroidota (former Bacteroidetes) (≅23%), including the genera *Bacteroides* and *Prevotella*, followed by the phyla Actinomycetota (former *Actinobacteria)* (≅3%) and Verrucomicrobia (≅2%) [4,5].

Gut microbiota is part of a complex community, and they interact with each other and the host to modulate biological processes essential to health. The gut microbiota play central roles in the host, as they are involved in the following processes: (i) the maintenance of the endothelial cell barrier, providing intestinal microvasculature, cell renewal, and wound healing, in addition to regulating mucus-renewal properties [6,7] or the remodeling of mucin glycosylation and the maintenance of intestinal epithelial tissue tight junctions through the activation of peptidoglycan-signaling Toll-like receptor 2 (TLR2) [2,3,8,9]; (ii) immunomodulation, influencing the development of the systemic immune system, as intestinal microorganisms contribute to expand T cells and innate lymphoid cells, to increase B cells for Immunoglobulin A (IgA) production, and in response to pathogens, to modulate the resident macrophages of the gut [2,3,8,9]; (iii) antimicrobial protection against pathogens through their structural components, metabolites, and the synthesis of antimicrobial proteins, including local immunoglobulins that control the growth of pathogenic bacteria [2,3,9]; and (iv) the generation of metabolites that influence the health of the host [5,9]. Consequently, the gut microbiota composition may be associated with either an improvement in human health or an imbalance (known as dysbiosis). Studies suggest that changes in the diversity and relative abundance of the microbiota and microbial metabolism are associated with various physical, neurological, and psychiatric disorders [10].

**Figure 1:**
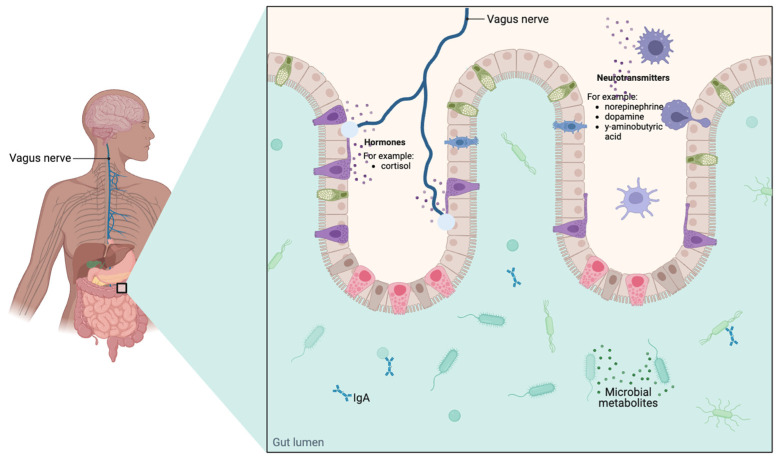
Possible communication pathways between the human microbiota and the brain.

The gut microbiome has long been considered an integral part of gut–brain communication and a microbiome–gut–brain axis [11,12,13,14] (Figure 1), since intestinal microorganisms communicate with the central nervous system (CNS) through neural (as is the case with microorganisms that synthesize neurotransmitters, for example, γ-aminobutyric acid (GABA), norepinephrine, and dopamine), endocrine (cortisol, for example), and immunological (cytokines, for example) signaling channels, playing an essential role in brain and behavioral changes [15].

Diet is a crucial factor influencing the gut microbiota, indicating a complex and bidirectional relationship between them. Therefore, the absorption and metabolism of nutrients can be influenced by the composition of the gut microbiota, which significantly impacts the host’s physiology [16]. The number of studies that relate food intake to the modulation of the intestinal microbiota has increased, and there is evidence that diet has a considerable effect on the gut microbiota, with an interaction between nutritional and microbial dynamics [17].

The gut microbiome can modulate nutrient metabolism upon dietary intake and produce many metabolites to interact with the host in various ways, including regulating glucose and lipid metabolism pathways, influencing the differentiation and function of immune cells, affecting insulin sensitivity, and so on. An overwhelming amount of human and animal data provides strong evidence of the crucial role of the gut microbiome and its metabolites in the occurrence and development of many metabolic diseases [18].

Oilseeds, also called edible nuts, are essential sources of fatty acids, phytosterols, minerals, and vitamins [19,20]. Pistachios and peanuts are two important oilseeds (considered edible nuts with a low-fat content when compared to other nuts), which have a nutritional profile rich in protein, healthy fats, and fiber, as well as a variety of vitamins, minerals, and phenolic compounds that have significant health benefits [21,22]. Because oilseeds have different chemical compositions in terms of bioactive compounds, the intake of each oilseed may have a different impact on the gut microbiota [23,24].

**Figure 2:**
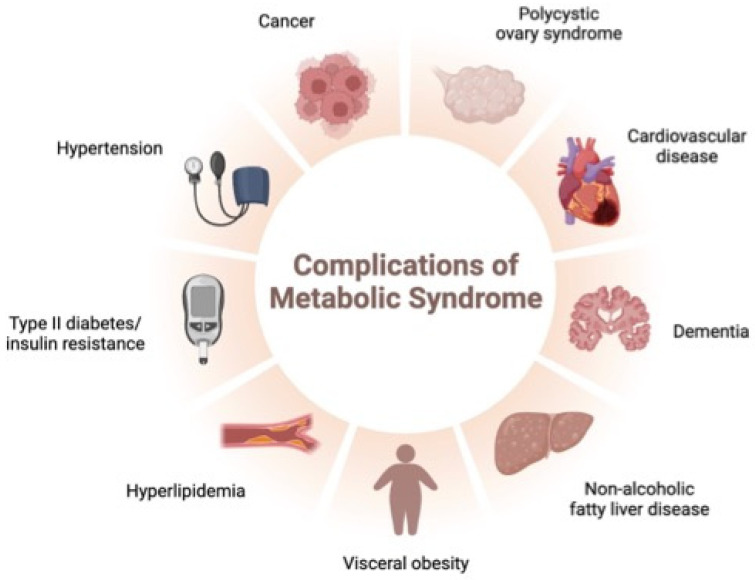
An overview of the complications of metabolic syndrome (MetS).

MetS is a pathological condition characterized by the presence of at least three of the following five risk factors: (i) abdominal obesity with a waist circumference ≥ 88 cm in women and ≥102 cm in men; (ii) high triglycerides ≥ 150 mg/dL; (iii) high-density lipoprotein cholesterol (HDL-c) < 50 mg/dL in women and <40 mg/dL in men; (iv) high blood pressure systolic ≥ 130 mmHg or diastolic ≥ 85 mmHg; and (v) fasting blood glucose ≥ 100 mg/dL [25,26,27] (Figure 2). Changing the diet and adding foods rich in bioactive compounds or isolated chemical compounds have often been associated with an improvement in these factors, which results in an improvement in metabolic syndrome [28]. Furthermore, one of the changes that occurs with these bioactive compounds is the improvement of the gut microbiota, which has been associated with an improvement in risk factors for metabolic syndrome [29].

As the consumption of nuts has been increasingly frequent in the human diet, it is evident that this consumption plays an essential role in human health by influencing the gut microbiota composition [16]. Thus, this review aimed to describe the effects of the ingestion of peanuts and pistachios on the gut microbiota and the possible roles this microbiota play in human health. We used the association of terms “metabolic syndrome”, “obesity”, “hyperlipidemia”, “dyslipidemia”, “hypertension”, “high blood pressure”, “diabetes”, and “pistachio” or “peanut” in the English language, in search of the PubMed, Scopus, and Web of Science databases in March 2023. We also checked the supporting references of the selected articles. The inclusion criteria used were the following: (1) in vivo study (with animals and/or humans); (2) intervention with peanut or pistachio alone or their fractions; (3) outcomes related to MetS or its risk factors (obesity, hypertension, diabetes, and dyslipidemia); (4) research studies that have focused on the composition of the intestinal microbiota; and (5) publication in English.

## 2. Peanuts

### 2.1. General Characteristics of Peanuts

Peanut is a *Magnoliopsida* (*Dicotyledoneae*) plant belonging to the *Fabaceae* (*Leguminosae*) family and the genus *Arachis*, originating in South America. Currently, 80 species are identified within the genus *Arachis*; however, the only cultivated species is *Arachis hypogaea* L. [30].

*Arachis hypogaea* L. is an annual plant that grows approximately 30–50 cm high. It has alternate, pinnately compound leaves with four leaflets (two opposite pairs; no terminal leaflet), each 1–7 cm long and 1–3 cm wide. The plant’s flowers appear in axillary groups above the ground. After pollination, a short, thick stem at the base of the flower, called a gynophore, grows downward and penetrates the soil such that the fruiting body develops entirely underground. Each seed is covered with a thin layer (film), followed by the epicarp (known as the peanut husk) [31].

### 2.2. Global Production

Peanuts are the fifth most produced oilseed, with 50 million tons in 2022/2023 crop production [32,33,34], and the world’s largest producers are China (37%), India (13%), Nigeria (9%), and the U.S. (5%). Peanuts are consumed differently worldwide, depending on habits, customs, and food culture. They are considered a complete and essential food source in Asian, African, and American countries and an important source of nutrients in the fight against malnutrition in places where protein-energy malnutrition persists and access to animal protein is more difficult or expensive. They are consumed in various ways, ranging from whole grain products, peanut butter, groceries, granola bars, and breakfast cereals to adding peanut flour or oil in food preparations [35,36,37].

### 2.3. Nutritional Profile and Potential Health Benefits

In terms of proximate composition, peanuts have water, ash, total carbohydrate, and dietary fiber contents of about 6, 2, 20, and 8 g/100 g, respectively, corresponding to a caloric value of 500 to 600 calories, depending on the fat content of the products (Table 1) [38]. In the proximal composition of peanuts, the contents of proteins and lipids are the main components [32,39,40,41]. As for micronutrients, peanuts contain 690, 380, 171, 89, 10, 4.43, 2.55, and 1.69 mg/100 g of potassium, phosphorus, magnesium, calcium, sodium, zinc, iron, and manganese, respectively [32].

The nutritional quality of peanuts is directly related to their lipid content (50 g/100 g), which is composed of saturated (~6 g/100 g), monounsaturated (~25 g/100 g), and polyunsaturated (~15 g/100 g) fatty acids [32], an oleic/linoleic acid ratio higher than 2 [52], and the presence of vitamin E classified as tocopherols (8.33 mg/100 g) [42,43]. In addition, the composition of fatty acids is important, mainly oleic (C18:1) and linoleic (C18:2) acids, constituting ~80% of peanut fatty acids. The remaining 20% of fatty acids are composed of palmitic acid (C16:0), stearic acid (C18:0), arachidic acid (C20:0), gadoleic acid (C20:1), behenic acid (C22:0), and lignoceric acid (C24:0) [53]. The high oleic/linoleic acid ratio is the most desired oilseed quality characteristic as it has a longer shelf life and health benefits for consumers [54], such as lowering serum cholesterol levels, suppressing tumorigenesis, and combating inflammatory diseases [55].

In addition, the nutritional quality of peanuts can also be related to their content of high-quality proteins (~25 g/100 g), which have a combination of essential amino acids (leucine, isoleucine, valine, lysine, methionine, tryptophan, phenylalanine, threonine, and histidine) that is closer to the combination found in human tissues [56]. Although the amino acid composition of peanuts varies significantly by plant variety and location, peanuts contain all twenty amino acids in varying proportions and are a source of arginine [57,58]. Abdualrahman [59] reported that raw peanuts contain 19.68 g/100 g glutamic acid, 10.07 g/100 g aspartic acid, 1.01 g/100 g methionine, 1.00 g/100 g cystine, 4.44 g/100 g glycine, 4.55 g/100 g alanine, 5.17 g/100 g valine, 317 g/100 g threonine, 3.82 g/100 g lysine, 6.10 g/100 g phenylalanine, 7.31 g/100 g leucine, 4.22 g/100 g isoleucine, and 13.31 g/100 g arginine.

Considering their chemical composition, peanuts are known as a functional food owing to their antitumor [60] and hypocholesterolemic [61] effects, as well as their cardiovascular protective properties [62]. However, the mechanisms by which peanut consumption confers these benefits are not fully understood and may be related partly to the physiological effects of the nutrients and bioactive compounds [63,64] and the role of the gut microbiome.

### 2.4. The Impact of Peanuts on the Gut Microbiota and Its Relationship with the Occurrence of MetS Risk Factors

Peanuts are a rich and diverse source of chemical compounds, making them an excellent option for promoting beneficial health effects [21]. Table 2 shows a summary of in vivo studies reporting the effects of peanuts on biochemical and physical parameters and the gut microbiota profile. 

#### 2.4.1. The Microbiota Improvement 

Peanut by-products also demonstrate microbial regulatory effects. In a study by Xiang et al. [66], the effect of the administration of peanut husk extract (80 mg/kg peanut husk extract) was studied in mice (n = 60) with T2DM (6 weeks) and compared to the treatment with metamorphine (a drug commonly used to treat T2DM). Regarding the intestinal microbiota of mice, the study demonstrated the presence of 9 phyla, 13 classes, 14 orders, 20 families, 22 genera, and 10 species. No *Actinomycetota* were detected, and a decrease in the ratio of *Bacillota* to *Bacteroidota* was observed, allowing the gut microbiota of mice with T2DM to recover to normal levels [66].

Xu, Lv, Wang, Lu, Ye, Zhu and Liu [69] demonstrated that the administration of 300 mg/kg peanut husk extracts for 12 weeks to ApoE^−/−^ mice significantly altered the composition of the gut microbiota by decreasing the amount of *Bacillota* and increasing the abundance of *Bacteroidota*, which could regulate the balance of the gut microenvironment. This study also demonstrated that peanut husk extract could increase the number of bacteria producing short-chain fatty acids, especially *Roseburia*, *Rothia*, *Parabacteroides*, and *Akkermansia*, while decreasing *Bilophila* and *Alistipes*. The abundance of *Akkermansia* in the intestine has been related to the secretion of glucagon-like peptide-1-inducing protein acting on glucose homeostasis and regulating intestinal microbiota [71].

In addition, the administration of peanut husk extract also significantly increased the abundance of *Parabacteroides distasonis* in the gut microbiota [69]. The *P. distasonis* bacterium may act as a probiotic by affecting intestinal bile acid metabolism, decreasing obesity (especially weight gain), and improving glucose and lipid metabolism [72]. In addition, the membranous components of *P. distasonis* may reduce the level of pro-inflammatory factors and increase specific antibodies to stabilize the intestinal microbiota [73].

Other microorganisms that are associated with T2DM are *Bifidobacterium pseudolc* and *Parabacteroides distasor*. In a study by Xiang, Wu, Osada, Yoshida, Pan and Qi [66], a reduction in the amount of *B. pseudolc* and *P. distasor* and an improvement in clinical symptoms, such as a reduction in fasting blood glucose and body weight (*p* < 0.001), were observed. Diabetes induced by a high-fat diet reduces gut integrity and increases endotoxemia by translocating bacterial lipopolysaccharide (LPS) from the gut microbiota into the blood. As explained above, the bacterial LPS induces low-grade inflammation and insulin resistance in the CNS and peripheral tissues [74].

Among the phytochemicals present in peanuts, the oleic acid content appears to be a major contributor to the regulation of intestinal microbiota. Peanut oil with high oleic acid content (providing up to 80% of the fatty acid composition) is also rich in monounsaturated fatty acids and minor bioactive phytochemicals, such as polyphenol phytosterols and vitamin E [65]. Zhao, Shi, Wang and Zhou [65] administered peanut oil rich in oleic acid to mice (n = 48) with MetS induced by high fructose for 12 weeks. These authors related that the peanut oil supplementation suppressed body weight gain (~360 and 420 g for the peanut oil diet group and high-fat diet group, respectively), improved the HDL/LDL ratio (~1.8 and 1.3 for the peanut oil diet group and high-fat diet group, respectively), and improved the process of insulin resistance in mice. Furthermore, 16S rDNA sequencing confirmed that the supplementation prevented the dietary disruption of the gut microbiota and promoted the proliferation of the phyla *Bacillota*, *Bacteroidota*, and *Actinomycetota* [65].

#### 2.4.2. The Role of the Metabolome

Previous studies have discussed how the gut microbiota attenuates MetS and its risk factors by modulating the intestinal microbiota [75]. These effects may be mediated in part by the metabolome, especially by the presence of branched-chain amino acids (BCAAs) [76], as many bacterial species can regulate the biosynthesis, transport and metabolism of BCAAs [77]. Pedersen, Gudmundsdottir, Nielsen, Hyotylainen, Nielsen, Jensen, Forslund, Hildebrand, Prifti and Falony [76] found that *Prevotella copri* and *Bacteroides vulgaris* are the main species driving the association between BCAA and biosynthesis and insulin resistance. In addition, Liu, et al. [78] observed that *Bacteroides thetaiotaomicron* reduced the concentrations of BCAAs and decreased diet-induced body weight gain and adiposity in mice.

Although it does not yet appear consistent in the literature due to the difficulty of demonstrating the same effect in humans (where there is a large interpersonal variation), there is a tendency to associate the decrease in the ratio of *Bacillota* to *Bacteroidota* with the occurrence of obesity, T2DM, high-fat diets, and consequently MetS. In these disorders, there may be a decrease in the abundance of *Bacteroides* in the gut and an increase in the abundance of *Bacillota*, resulting in a decrease in the ratio [79,80]. This result was observed in two animal model studies [66,67], which used peanuts in the diet but not in the human intervention with peanut consumption [70] (Table 1). Wang et al. [70] reported the difficulty of associating results from animal models with human models due to the individual gut microbiota configuration modulated by the host metabolism and altered by the individual response to the intervention.

#### 2.4.3. The Impact of Lipopolysaccharides

**Figure 3:**
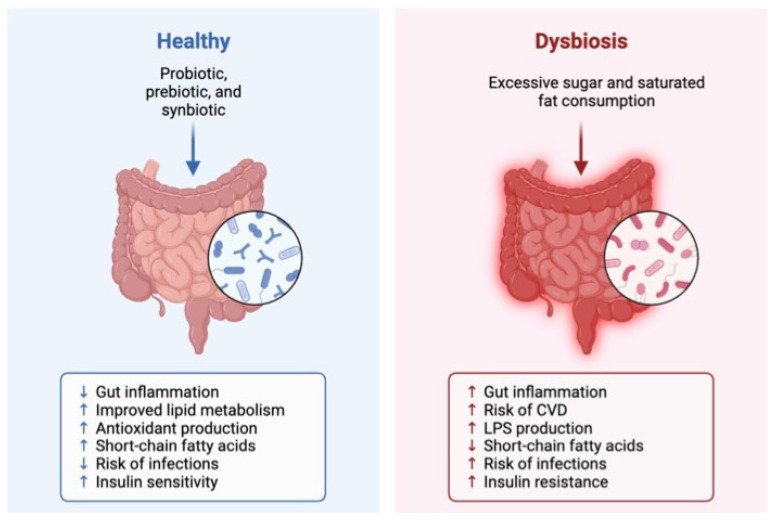
Scheme indicating healthy microbiota and dysbiosis microbiota.

Dysbiosis, an imbalance in the composition and function of bacteria that inhabit the intestine (Figure 3), caused by high-fat diets, obesity, and T2DM, among others, increases the blood levels of LPS produced by Gram-negative microorganisms [81]. Therefore, the ingestion of peanuts and their by-products decreases the presence of Mucispirillum [66] and *Lachnospiraceae* [65], which are associated with LPS. Inflammation [82,83] induces systematic inflammation in the peripheral tissue caused by this metabolite by activating Toll-like receptor 4 (TLR4) signaling [84].

#### 2.4.4. The Global Impact of Peanut Consumption

**Figure 4:**
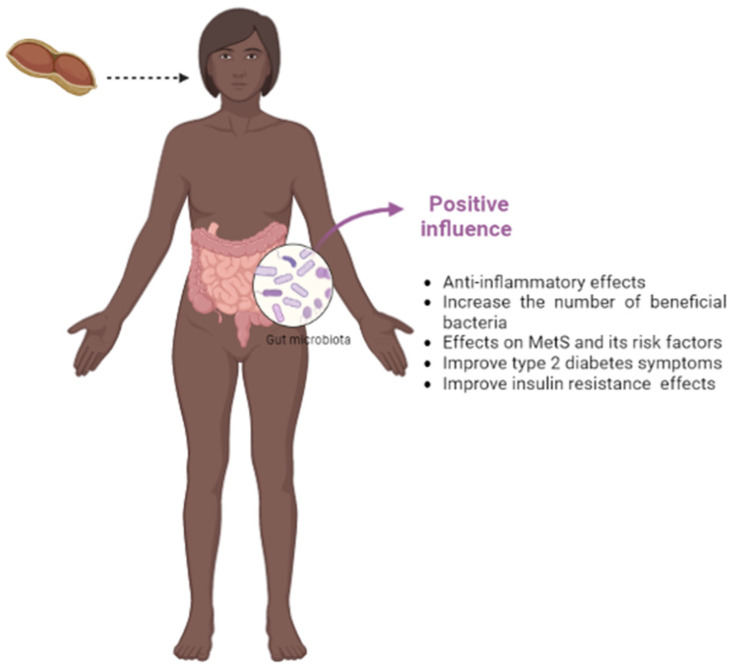
An overview of the possible beneficial effects associated with peanut consumption.

Studies have shown that peanut intake in in vivo models receiving a high-fat diet or a normal diet after the induction of obesity or type 2 diabetes (T2DM) helped improve clinical symptoms, such as obesity (especially body weight) and lipid and glucose metabolism, among others [65,66,67,70]. These results are important because metabolic syndrome (MetS) is of major concern among public health agencies (Figure 4).

Although both peanuts and their by-products appear to show effects on MetS and its risk factors (especially body weight and glucose and lipid metabolism) in animal studies by regulating the gut microbiota, the effects need to be further confirmed in human studies.

## 3. Pistachios

### 3.1. General Characteristics of Pistachios

Pistachios (*Pistacia vera* L., *Anacardiaceae* family) have been a part of the human diet since prehistoric times and were consumed by past civilizations [85]. These oilseeds are ripe in late summer or early autumn, their shells turn pink, and their inner shells naturally divide along their sutures. The pistachio tree grows up to 10 m tall and is a sun- and saline soil-tolerant desert plant. The pistachio is covered with a hard, whitish, and thick outer shell, which serves as protection and represents about 50% of its weight. The seed has a thin husk and light green pulp with a distinct flavor [86].

### 3.2. Global Production

The total world production of pistachios in 2022/2023 was estimated at 0.782 milion tons, of which approximately 51% was produced in the United States (U.S.), followed by Turkey (26%), Iran (13%), Syria (6%), and the European Union (3%) [34,87]. Pistachios are sold mainly in their shells, usually roasted and salted to be consumed as snacks, although the kernels can also be used in confectionery and cooking or to make other diverse products, such as pistachio paste [88] or milk [46].

### 3.3. Nutritional Profile and Potential Health Benefits

In terms of proximal composition, pistachio has a fat, protein, and fiber content of approximately 47.4, 20.4, and 10 g/100 g, respectively, corresponding to a caloric value of 580 calories (Table 1). Lipids consist mainly of monounsaturated (~23.3 g/100 g) and polyunsaturated (14.4 g/100 g) fatty acids, and small amounts of saturated fatty acids (~5.9 g/100 g) in the major fatty acids such as oleic acid and linoleic acid. Roasted pistachios have a protein digestibility-corrected amino acid score (PDCAAS) of 81, which is higher than raw pistachios [46]. The digestible indispensable amino acid (DIAAs) values for raw and roasted pistachios were 86 and 83, respectively [89].

In addition, pistachios can be considered a source of at least 15 different micronutrients, according to the U.S. Food and Drug Administration (FDA) (providing more than 10% of the Recommended Daily Value (RDV) per ounce (28.5 g) serving) or in the European Union (EU) (providing at least 15% of the Nutrient Reference Value per 100 g) according to the limits of the Nutrition and Health Claims Regulation (NHCR) [47,48]. Pistachios contain copper (~1.3 mg/100 g), manganese (~1.2 mg/100 g), vitamin B6 (~1.7 mg/100 g), thiamine (~0.87 mg/100 g), potassium (~1020 mg/100 g), phosphorus (~490 mg/100 g), vitamin E (~2.86 mg/100 g) and K (phylloquinone) (~0.0013 mg/100 g), riboflavin (~0.16 mg/100 g), folate (~51 mg/100 g), magnesium (~121 mg/100 g), iron (~3.92 mg/100 g), zinc (~2.2 mg/100 g), and selenium (~0.007 mg/100 g). In addition, pistachios contain carotenoids, such as lutein and zeaxanthin (xanthophyll carotenoids) (~2.9 mg/100 g) and beta-carotene (~0.305 mg/100 g), total phenolic compounds (~1677 mg G.A.E./100 g) consisting of isoflavones (~159 mg/100 g) and tocopherols (~20.6 mg/100 g), and chlorophylls (sum of a+b) (~1500–3800 µg/100 g) [49,50,51,90,91,92].

Pistachio is considered one of the world’s most consumed nuts due to its nutritional properties, and several international associations recommend its regular consumption [93]. Compared to other nuts, pistachios have a balanced nutritional profile with less fat (monounsaturated and polyunsaturated fatty acids) and more protein, fiber (both soluble and insoluble), potassium, phytosterols, γ-tocopherol, vitamin K, and xanthophyll-type carotenoids. [94]. Pistachios are also known for their high antioxidant potential [95].

### 3.4. The Impact of Pistachio on the Gut Microbiota

Table 3 presents a summary of in vivo studies reporting the impact of pistachio consumption on biochemical and physical parameters and the gut microbiota profile. 

#### 3.4.1. The Microbiota Improvement 

Ukhanova, Wang, Baer, Novotny, Fredborg and Mai [98] evaluated the composition of the gut microbiota of 16 volunteers who consumed 85 g/day of pistachios for 18 days. The pistachio consumption affected the composition of the gut microbiota by increasing the number of beneficial butyrate-producing bacteria, such as *Faecalibacterium prausnitzii* and *Eubacterium rectale*/*Roseburia* spp., which was higher compared than that in other edible nuts. According to a study by Mandalari, et al. [99], simulated human digestion showed that pistachios (raw pistachios, roasted salted pistachios, and muffins made from raw pistachios) can be regarded as a prebiotic agent, releasing compounds, such as polyphenols, tocopherols, and lutein, after gastric and enteric fermentation, which can potentiate the growth of non-pathogenic intestinal bacteria while inhibiting the growth of pathogenic species.

A study conducted by Yanni, Mitropoulou, Prapa, Agrogiannis, Kostomitsopoulos, Bezirtzoglou, Kourkoutas and Karathanos [96] aimed to investigate the effect of pistachio administration for four weeks on the composition of the gut microbiota of rats with type 1 diabetes mellitus (T1DM). The pistachio supplementation, even with no effect on body weight and the plasma lipid profile, significantly increased the populations of lactobacilli and bifidobacteria in the jejunum, ileum, and caecum, decreased the *Enterococci*, and normalized the microbiota in all examined intestinal regions of animals. Lactobacillus and Bifidobacterium species are capable of producing folate, which, when absorbed by the intestine, plays a crucial role in the synthesis of 5-methyltetrahydrofolate. This compound acts as a methyl donor, promoting the methylation of DNA and RNA in the form of N6-methyladenosine (m6A). This methylation is essential to ensure the healthy development of intestinal tissue [100]. 

Terzo, Mulè, Caldara, Baldassano, Puleio, Vitale, Cassata, Ferrantelli and Amato [97] evaluated whether replacing 20% of the caloric intake of a high-fat diet with pistachios (180 g/kg diet) prevented inflammation and associated dysbiosis in mice. The authors reported that the high-fat diet supplemented with pistachios significantly increased the abundance of healthy bacterial genera, such as *Parabacteroides*, *Dorea*, *Allobaculum*, *Turicibacter*, *Lactobacillus*, and *Anaeroplasma*, and decreased pro-inflammatory bacteria, such as *Oscillospira*, *Desulfovibrio*, *Coprobacillus*, and *Bilophila*. 

Although the gut microbiota was not studied, Hernández-Alonso, Cañueto, Giardina, Salas-Salvadó, Cañellas, Correig and Bulló [94] showed, using a randomized cross-over study (n = 39), that certain metabolites in urine (hippurate, p-cresol sulfate, dimethylamine, cis-aconitate, creatinine, and trimethylamine N-oxide) associated with gut microbiota metabolism from pistachio consumption were associated with insulin resistance and T2DM. In this study, these metabolites were significantly modulated, resulting in an improvement in the homeostasis of the individuals. Interest in nutritional metabolomics arising from the gut microbiota has increased due to their significant role in the diagnosis and prognosis of various diseases. In addition to host metabolism, the complex gut microbial ecosystem produces a variety of metabolites that may play important roles in human health.

Edible seeds, such as pistachio, have prebiotic effects in the gut, which in turn are fermented and partially absorbed during the digestive process in the intestine. Unabsorbed complex polyphenols are bioactivated in the colon by the microbiota. The active metabolites of polyphenols are found in the host’s blood and can, therefore, positively affect metabolism and human health [50]. Furthermore, these metabolites from phenolic compounds influence the growth of certain microbial species [101], and it has been reported that diets rich in monounsaturated fatty acids increase fecal bifidobacteria in volunteers. Dietary fiber provides a substrate for microbial fermentation in the gut and facilitates the maintenance or selection of healthy microbiota composition. Therefore, pistachios can be proposed as a food with prebiotic properties that have significant potential for health maintenance via microbiota regulation [98,102].

#### 3.4.2. The Global Impact of Pistachio Consumption

Clinical research studies conducted with human participants have shown that pistachio consumption results in a positive influence on oxidation biomarkers and antioxidant defenses [103,104,105]. 

**Figure 5:**
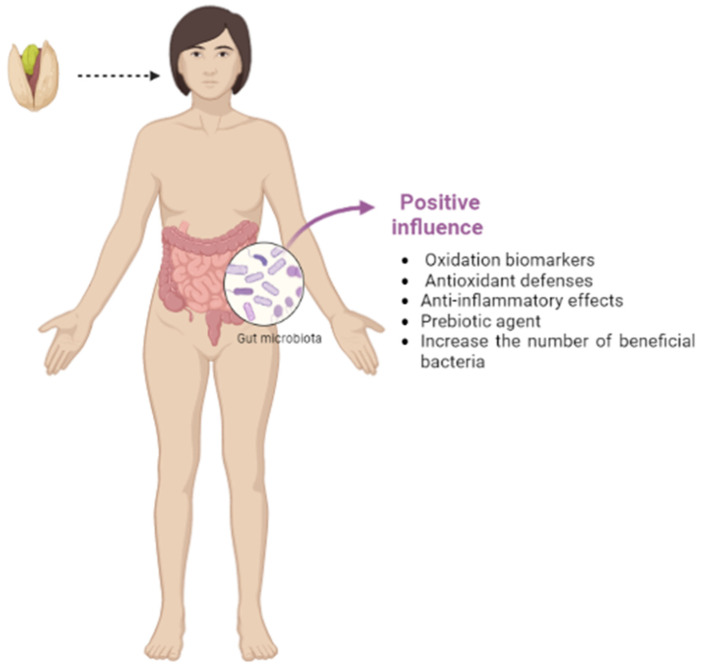
An overview of the possible beneficial effects associated with pistachio consumption.

The anti-inflammatory effects of pistachios have also been reported in several clinical trials, evaluating changes in inflammation biomarkers, such as C-reactive protein, tumor necrosis factor (TNF), and adhesion molecules (ICAM-1 and VCAM-1) [105,106,107]. In addition, pistachio consumption can positively alter the composition of the gut microbiota [96,97,98], and this modulation can attenuate MetS and its risk factors (obesity, T2DM, hypertension, hypercholesterolemia, etc.) (Figure 5), which has been demonstrated by several authors [103,105,106,108], although some of these works were not included in this review because they are not works that study changes in the intestinal microbiota.

Thus, changes in the microbiota composition can be attributed to the different components of pistachios, such as fatty acids, flavonoids, or fibers, which may influence the bacterial fecal microbiota by increasing the microorganisms responsible for the production of butyrate, possibly due to their prebiotic effect. However, further studies are needed to verify the potential of pistachios in diabetes and obesity, as well as randomized controlled studies with specific groups of volunteers.

## 4. Findings, Limitations, and Areas for Future Research

This literature review showed that most of the evidence regarding the effects of peanuts and pistachios on gut microbiota and metabolic syndrome is related to studies conducted in animal models, demonstrating that there may be a positive correlation between the intake of peanuts or pistachios and improvement in microbiota with impact on MetS and its risk factors. However, it should be noted that one of the limitations of this review is that the extrapolation of these results to human beings is still incipient and requires clinical studies of the randomized controlled trial (RCT) type. Only two of the nine studies in the literature found for the formulation of this review are R.C.T.s, one for peanuts and one for pistachios.

In this context, there are still gaps that need to be filled for dietary recommendations by health professionals. When it comes to peanuts, the animal studies used doses between 0.5 and 30% in the animal feed that was available *ad libidum* [65,66,67,68,69], while the human study used a portion of 56 g/day [70]. When it comes to pistachios, animal studies used doses of 8.5% [96] and 18% [97] in the animal feed that was available ad libitum, while the human study used 45 and 60 g/day [98]. For both peanuts and pistachios, human studies have tested larger amounts than the recommended serving sizes for nut and almond intake, which is typically one serving daily (30 g/day) or 4–6 servings per week [109]. In addition to the large variation in the amount administered in animal studies, when it comes to translating into daily portions recommended for humans, problems arise regarding this “overdose”, which may not cause harm to the health of the volunteer but leads to an overestimation of the outcomes (final results) of the research, in addition to being often impractical. Furthermore, this intake of nuts can result in high energy intake that will directly affect the outcome of studies, especially those related to MetS. Studies in animal models control the weekly feed intake, while studies in humans control intake using a food frequency questionnaire or 24 h dietary recall, which may explain the increase in energy intake. More effectively, Ukhanova, Wang, Baer, Novotny, Fredborg and Mai [98] seem to have reduced the errors embedded in this issue by providing weekend meals alongside treatment products with instructions for home consumption.

When carrying out RCTs, comparison groups can be adopted, which can be groups without intervention in conventional care, in addition to placebo groups and/or using another type of intervention [110]. Clinical food intake trials, where the intake of food is compared to a group of volunteers without intervention, can be carried out, as was the case with the pistachio RCT [98]. However, some points are still ethically conflicting since when inviting volunteers to participate in research, the researcher reports the benefits that this research can bring, and when dealing with foods with functional potential, the beneficial effects on human health are portrayed. Once the volunteers are aware of the benefits (even if it is being tested by research) and also, if the food sample being evaluated is available and accessible to the population, there may be an undue and uncontrolled intake of the food to be tested by research volunteers even though they were randomized into the non-intervention groups.

Another way to solve this problem is the use of “placebo” groups, as discussed by Wang, Zhang, Wang, Huang, Zhao, Malik, Liu, Sun, Lin and Chen [70], who evaluated peanuts against a control group that ingested one isocaloric rice bar of 82 g/day. In these cases, the situation is a little more challenging, as the objective of adopting “placebo” groups is precisely to achieve an intervention that is indistinguishable from the treatment to be tested (in this case, pistachio) in terms of physical appearance, taste, or odor, but which does not include the possible primary/secondary outcome tested [110]. Placebo groups are difficult to implement in the food sector since every addition or removal of an ingredient alters the traditional food. In this sense, it is an arduous task to isolate in clinical trials the placebo effect of the study of food intake, mainly due to the awareness of the act of eating of the individuals used in the studies [111]. The suggestion that has been put forward in these cases is the adoption of questionnaires on experiences together with biochemical and/or anthropometric markers to find a global index that allows a general assessment using the markers studied [112]. 

The sample size of the peanut RCT is still small, and therefore, as recommended by the authors themselves, a larger study sample would provide greater statistical power and thus improve the ability to predict the need for daily intake to result in health benefits [70]. Finally, although the RCT that studied the daily pistachio consumption used a larger population, clarifications regarding the increase in microorganisms producing short-chain fatty acids (especially butyrate) in the microbiota is still needed. Future studies need to be of experimental design with very well-defined primary and secondary health-related outcomes [98]. 

Last but not least, it is important to highlight that there is a qualitative/quantitative difference between the animal and human microbiota, and therefore many studies have used germ-free animals with fecal transplants from humans [113]. The studies reported in this literature review for peanuts and pistachios did not use this model. Therefore, there may be differences in the composition and abundance of the microbiota reported by these authors. What is reported in this subcategory does not diminish the importance of these works but highlights the need for more specific pre-clinical studies related to this issue.

## 5. Conclusions

Peanuts and pistachios are rich in protein, unsaturated fatty acids, vitamins, and polyphenols, which are food components that have been shown to benefit human health. This review demonstrated that ingesting peanut and pistachio seeds can beneficially modulate the intestinal microbiota, enhancing the growth of beneficial intestinal bacteria, especially those producing short-chain fatty acids, enabling favorable health effects. In addition, studies have shown that the modulation of the gut microbiota via peanut consumption seems to attenuate MetS and its risk factors (obesity, type 2 diabetes, hypertension, hypercholesterolemia, etc.). At the same time, pistachio’s potential still needs to be further demonstrated through in vivo studies. In future research, it is essential to adopt appropriate control groups, such as non-intervention, conventional, or placebo groups, to avoid bias in the results of randomized clinical trials (RCTs). Furthermore, expanding sample sizes is essential to improve statistical power and more accurately predict health benefits. Furthermore, additional studies that address the difference between animal and human microbiota are needed, highlighting the importance of more specific investigations, especially in pre-clinical models.

## Figures and Tables

**Table 1:** Nutritional components of peanut and pistachio edible nuts.

Components	Peanut [32,38,42,43]	Pistachio [44,45,46,47,48,49,50,51]
Moisture (g/100 g)	6	4.5
Ash (g/100 g)	2	2.8
Protein (g/100 g)	25	20.4
Lipid (g/100 g)	50	47.4
Dietary fiber (g/100 g)	8	10
Carbohydrate (g/100 g)	20	14
Energy (kcal g/100g)	674	620
Potassium (mg/100 g)	690	1020
Phosphorus (mg/100 g)	380	490
Magnesium (mg/100 g)	171	121
Calcium (mg/100 g)	89	23
Sodium (mg/100 g)	10	
Zinc (mg/100 g)	4.33	2.20
Iron (mg/100 g)	2.55	3.92
Manganese (mg/100 g)	1.69	-
Saturated fatty acids (g/100 g)	6	6
Monounsaturated fatty acids (g/100 g)	25	23
Polyunsaturated fatty acids (g/100 g)	15	14
Tocopherols (mg/100 g)	8.33	20.6
Zeaxanthin (mg/100 g)	-	2.9
Beta-carotene (mg/100 g)	-	0.305
Total phenolic compounds (μmol TE/g DW)	16.2	1677

DW: dry weight.

**Table 2:** A summary of in vivo studies reporting the impact of peanut consumption on biochemical and physical parameters as well as the gut microbiota profile.

Food Intervention	Type of Study	Model	Main Results	References
High Oleic Acid Peanut Oil (HOPO)	Animal	Male Sprague Dawley rats fed for 12 weeks with 10% HOPO plus a high-fat diet and water containing 10% fructose	***Overall results:*** ↑ insulin sensitivity. ↓ liver TG, fat accumulation; plasma fasting insulin, HOMA-IR, TC, TG, and LDL levels. ***Gut microbiota results:*** ↑ Family level: *Clostridiaceae_1*, *Anaeroplasmataceae*, *Bifidobacteriaceae*, *Erysipelotrichaceae*, and *Planococcaceae;* genus level: *Olsenella*, *Peptoclostridium*, *Ruminococcaceae_UCG-009*, *Weissella*, *Bifidobacterium*, *[Eubacterium]_fissicatena_group*, *[Eubacterium]_coprostanoligenes_group*, *Ruminococcaceae_NK4A214_group*, *Clostridium_sensu_stricto_1*, *Ruminococcaceae_UCG-014*, and *Faecalibaculum.* ↓ Family level: *Lachnospiraceae*, *Micrococcaceae*, *Streptococcaceae*, and *Bacteroidaceae;* genus level: *Bilophila*, *Leuconostoc*, *[Eubacterium]_nodatum_group*, *Lactococcus*, *uncultured_bacterium_f_Coriobacteriaceae*, *Streptococcus*, *Rothia*, *[Ruminococcus]_torques_group*, *Bacteroides*, *Lachnoclostridium*, and *Blautia.*	Zhao, et al. [65]
Peanut skin extract (PSE) with doses of 10, 80, and 160 mg/kg per day for 6 weeks	Animal	Mice with type 2 diabetes mellitus (T2DM) induced by high-fat diet for 4 months until the mice presented >7 mmol/L blood glucose concentration, obesity, polydipsia, polyphagia, and polyuria.	***Overall results:*** ↑ glucose tolerance and insulin sensitivity. ↓ fasting blood glucose; liver, epididymal fat, heart, pancreas, and kidney weights; plasma TG and TC; pro-inflammatory cytokines in plasma and gene expression levels in adipose tissue; and LPS in the blood. ***Gut microbiota results:*** ↑ Cyanobacteria phyla. ↓ *Bacillota* to *Bacteroidota* ratio; *Bifidobacterium pseudolc* and *Parabacteroides distasor;* and Mucispirillum at the genus level. Actinomycetota and *Ruminococcaceae-6* were not detected.	Xiang, et al. [66]
High oleic peanut (D7) and peanut cv. Hanoch (HN)	Animal	Mice (male C57BL/6J) fed for 10 weeks with normal and high-fat diets plus peanut (4%)	***Overall results:*** ↑ plasma fasting glucose in HN; plasma TC and HDL in peanut groups; n-6/n-3 in liver tissue in peanut groups; and Srebp1C, PPARα, TNF, and iNOS gene expression in D7-group. ↓ AUC in peanut groups; plasma fatty acid, plasma TG, lipid fatty accumulation, and TG in the liver in D7-group. ***Gut microbiota results:*** ↑ diversity of bacteria in D7-group; *Prevotella* in D7-group; and *Bacillota* phyla in D7-group. ↓ *Pseudomonadota* (former *Proteobacteria*); *Deferribateres*; *Verrucomicrobia*; *Bacillota*/*Bacteroidota* ratio in peanut groups; and *Bacteriodetes* phyla in D7-group.	Bimro, et al. [67]
Peanut meal fermented by *Bacillus natto* with doses of 0, 0.3, 1.5, and 7.5 g/kg per day	Animal	Male Kunming mice (n = 90) fed by gavage of 0.1 mL/g body weight per day.	***Overall results:*** ↑ better growth and development; enhancement of learning and memory capacity; preventive role in antibiotic-induced dysbacteriosis; increased richness; and uniformity of the gut microbiota. ***Gut microbiota results:*** ↑ *Bacteroidota*; *Deferribacteres*; *Bacillota*; *Pseudomonadota*; and *Tenericutes.* ↓ *Rikenellaceae*_RC9_gut_group; *Peptoclostridium*; *Escherichia-Shigella*; *Lachnospiraceae*_UCG-001; *Parasutterella; Helicobacter*; *Enterobacter*; *Parabacteroides*; *Lachnospiraceae*_NK4A136_group; and *Bacteroides*.	Jiang, et al. [68]
Peanut skin extract (PSE) with doses of 150 and 300 mg/kg per day for 12 weeks	Animal	ApoE^−/−^ mice (C57BL/6J) fed 10% fat kcal per day	***Overall results:*** ↑ HDL-c content and IL-10 anti-inflammatory cytokine. ↓ plasma TC; LDL-c content; and pro-inflammatory cytokines TNF and IL-6. ***Gut microbiota results:*** ↑ *Roseburia*, *Rothia*, *Parabacteroides*, and *Akkermansia* ↓ *Bilophila* and *Alistipes*.	Xu, et al. [69]
56 g/day of peanuts divided into two portions: one packet within 1 h before lunch and one packet within 1 h before dinner	Human	Participants (n = 209) with central obesity and at least one other risk factor for MetS from a 12-week randomized clinical trial	***Overall results:*** ↓ body weight; waist circumference, and fasting blood glucose. ***Gut microbiota results:*** ↓ *Bilophila*; *Coprococcus_3;* and *Dorea.*	Wang, et al. [70]

AUC.: area under the curve; HDL: high-density lipoprotein; HOMA-IR: homeostatic model assessment of insulin resistance; IL-6: interleukin 6; IL-10: interleukin 10; iNOS: nitric oxide synthase; LDL: low-density lipoprotein; LPS.: bacterial lipopolysaccharide; PPARα: peroxisome proliferator-activated receptor alpha; Srebp1C: sterol regulatory element-binding protein 1; T2DM: type 2 diabetes mellitus; TG: triglycerides; TC: total cholesterol; and TNF-α: tumor necrosis factor-alpha. ↑ increase and ↓ decrease.

**Table 3:** A summary of in vivo studies reporting the impact of pistachio consumption on biochemical and physical parameters as well as the gut microbiota profile.

Food Intervention	Type of Study	Model	Main Results	References
8.5 g/100 g whole and fresh pistachio diet, including skin, except the shell. Fixed amount daily in the morning.	Animal	Male Wistar rats with T1DM induced with streptozotocin solution (40 mg/kg) (diabetic) and healthy animals. Duration 4 weeks.	***Overall results:*** Pistachio did not affect body weight or the plasma lipid profile. ***Gut microbiota results:*** ↑ bifidobacterial counts in fecal, jejunum, ileum, and caecum microbiota for healthy and diabetic rats; bifidobacterial counts in colon for healthy rats; lactobacilli count in fecal, ilium, and caecum microbiota for healthy and diabetic rats; *Turicibacter* and *Lactobacillus* genera in fecal microbiota for healthy rats; *Bifidobacterium* in fecal microbiota for diabetic rats; and *Romboutsia* levels for fecal microbiota for healthy and diabetic rats. ↓ lactobacilli count in colon microbiota for diabetic rats; enterococci counts in fecal, jejunum, cecum, and colon microbiota; *E. coli* population in fecal and colon microbiota of diabetic rats; *E. coli* population in jejunum and caecum microbiota for healthy and diabetic rats; *Enterobacteriacae* in ileum and cecum microbiota for healthy and diabetic rats; *Enterobacteriacae* and coliforms in jejunum microbiota for diabetic rats; Coliforms in ilium microbiota for healthy and diabetic rats.	Yanni, et al. [96]
Hyperlipidic diet with 20% of caloric intake replaced by pistachios (180 g/kg HFD) for 16 weeks	Animal	Mice (male C57BL/6J) fed for 4 weeks with a normal and high-fat diet	***Overall results:*** ↓ TNF- α; IL-1β; number and area of adipocytes, crown-like structure density, IL-1β, TNF-α, CCL-2 mRNA expression levels; liver: IL-1β e CCL-2. ***Gut microbiota results:*** ↑ Genus level: *Parabacteroides*, *Dorea*, *Allobaculum*, *Turicibacter*, *Lactobacillus*, and *Anaeroplasma*; ↓ Ratio *Bacillota/Bacteirodetes;* genus level: *Oscillospira*, *Desulfovibrio*, *Coprobacillus*, and *Bilophila*	Terzo, et al. [97]
Three treatment groups: (1) no nuts; (2) 1.5 servings/day of almonds or pistachios; (3) 3 servings/day of almonds or pistachios.	Human	Volunteers (n = 16) were recruited to participate in two separate randomized, controlled, cross-over studies with three 18-day feeding periods separated by an elimination period of at least 2 weeks.	***Gut microbiota results:*** ↑ Butyrate-producing bacteria. *Bifidobacterial;* α-diversity; proportions of the main phyla; and numbers of lactic acid bacteria and bifidobacteria were not affected.	Ukhanova, et al. [98]

T1DM: type 1 diabetes mellitus; TNF-α: tumor necrosis factor-alpha; IL-1β: interleukin 1β; HFD: Hyperlipidic diet, and CCL-2: chemokine (C-C motif) ligand 2. ↑ increase and ↓ decrease.

## Data Availability

Not applicable.
